# Growing up with Fragile X Syndrome: Concerns and Care Needs of Young Adult Patients and Their Parents

**DOI:** 10.1007/s10803-019-03973-7

**Published:** 2019-03-16

**Authors:** M. C. Van Remmerden, L. Hoogland, S. E. Mous, B. Dierckx, M. Coesmans, H. A. Moll, K. Lubbers, C. R. Lincken, A. M. Van Eeghen

**Affiliations:** 1 The Hartekamp Groupe, Care and Service Center for People with Intellectual Disabilities, Haarlem, The Netherlands; 2 ENCORE Expertise Center for Neurodevelopmental Disorders, Erasmus Medical Center, Room SP-1536, Wytemaweg 80, 3015CN Rotterdam, The Netherlands; 3 Intellectual Disability Medicine, Department of General Practice, Erasmus Medical Center, Rotterdam, The Netherlands; 4 ASVZ, Care and Service Center for People with Intellectual Disabilities, Sliedrecht, The Netherlands; 5 Department of Child and Adolescent Psychiatry/Psychology, Erasmus Medical Center–Sophia Children’s Hospital, Rotterdam, The Netherlands; 6 Department of Psychiatry, Erasmus Medical Center, Rotterdam, The Netherlands; 7 Department of General Pediatrics, Erasmus Medical Centre–Sophia Children’s Hospital, Rotterdam, The Netherlands; 8 ‘s Heeren Loo Zorggroep, Care and Service Center for People with Intellectual Disabilities, Amersfoort, The Netherlands

**Keywords:** Neurodevelopmental disorders, Fragile X syndrome (FXS), Qualitative research, Transition, ICF classification, Intellectual disability

## Abstract

Little is known about care needs of young adults with Fragile X Syndrome (FXS). Patient-driven information is needed to improve understanding and support of young adults with FXS. A qualitative study was performed in 5 young adult patients (aged 18–30), and 33 parents of young adults. Concerns and care needs were categorized using the International Classification of Functioning, Disability, and Health. Results indicated concerns on 14 domains for males, and 13 domains for females, including physical, psychological and socio-economical issues. In both groups parents reported high stress levels and a lack of knowledge of FXS in adult care providers. This study revealed concerns on various domains, requiring gender-specific, multidisciplinary transitional care and adult follow-up for patients with FXS.

## Introduction

Fragile X syndrome (FXS) is one of the most common heritable causes of intellectual disability (ID) with an estimated prevalence between 1 in 4000–7000 for the male population, and 1 in 8000–11,000 for the female population (Hunter et al. 2014). FXS is caused by a full mutation in the FMR1 gene on the X chromosome, caused by unstable expansion of the cytosine–guanine–guanine (CGG) repeat at the 5′untranslated region. A repeat extending beyond 200 repeats inhibits FMR1 transcription, resulting in loss or heavy reduction of the protein product FMRP, and hence the FXS phenotype (Pieretti et al. 1991).

Clinical features of male patients include developmental delay and dysmorphic features such as an elongated face, prominent forehead, large ears, and macro-orchidism (Ciaccio et al. 2017). The intelligence quotient (IQ) of males varies with a mean of 40–50 (Alanay et al. 2007; Merenstein et al. 1996). The behavioral phenotype in males can include attention deficit hyperactivity disorder (ADHD), autism spectrum disorder (ASD), anxiety, aggressive behavior and self-injurious behavior (Ciaccio et al. 2017). Manifestations in adult males are understudied (Schneider et al. 2013), and include seizures, movement disorders, gastrointestinal problems, hypertension, obesity and heart problems (Utari et al. 2010). Relatively low cholesterol levels have also been reported (Berry-Kravis et al. 2015). The behavioral phenotype seems to persist, although hyperactivity declines. Also, cognitive and adaptive functioning have been reported to decline (Fisch et al. 2002; Klaiman et al. 2014).

Females with FXS generally have milder symptoms due to presence of one normally FMRP-producing X chromosome, and studies on manifestations in both childhood and adulthood are limited. Approximately 53–71% of females with full mutations have IQs in the borderline or intellectual disability range (de Vries et al. 1996; Hagerman et al. 1992). Females with the full mutation are more likely to present with social anxiety, shyness, social avoidance, withdrawal, language deficits, mood lability, and depression (Bennetto et al. 2001; Hagerman et al. 1992). In adult females, most frequent manifestations include gastrointestinal symptoms, hypertension and obesity. Neurological problems can occur, but less frequently compared with males with FXS (Utari et al. 2010). Specific research on mental health in adult female patients is lacking, although clinical experience indicates that the behavioral phenotype persists in adult life.

An American healthcare guideline is available for pediatric and adolescent patients with FXS (Hersh et al. 2011). The American Academy of Pediatrics advises periodic health assessments throughout childhood and adolescence, including a full developmental and educational assessment, an evaluation of the cognitive level and a medical screening for medical issues associated with FXS. In this guideline, the adult population is only mentioned briefly. No specific guideline is available for the transitional period or for adult patients with FXS.

The transitional age is usually defined as the life span between 15 and 25 years, and is a stage of physical and psychological development in which many transitions take place, involving daytime occupation, living arrangements and relationships. Transitional healthcare is defined as “the purposeful, planned movement of adolescents and young adults with chronic physical and medical conditions from child-centred to adult-oriented healthcare systems” (Blum et al. 1993), and takes place during the same period. This healthcare transition is often problematic, as specialized multidisciplinary healthcare for adults with rare genetic disorders is often unavailable (Van Lierde et al. 2013). Loss to follow-up often occurs, leading to poor health outcomes (Goossens et al. 2016). Literature on transitional health care for patients with FXS are limited, even though difficulties in access to a qualified primary care provider and specialty services have been reported (Wheeler et al. 2018). Also, some factors associated with loss to follow-up have been identified (Kidd et al. 2017). In patients with ASD (Friedman et al. 2013), epilepsy (Borlot et al. 2014; Geerlings et al. 2015), and ID (Young-Southward et al. 2017b), comorbidity all present in FXS, an unsuccessful transition leads to poor outcomes on multiple domains. Areas of concern in young adults with ID are physical and mental health and well-being, with obesity, sexual health, social relationships, employment and independent living being areas of concern (Young-Southward et al. 2017a; Luftig and Muthert 2005). Studies reporting on the experience of parents of children with ID of their child’s transitional age phase showed that most parents were not satisfied with information provision, coordination of care, access to adult health care providers, and knowledge of the health care provider (Griffith et al. 2011; Udwin et al. 1998).

Up to now, little is known about the worries and the healthcare needs of young adult male and female FXS patients and their parents, and no guidelines for adults with FXS are available, even though patients remain affected in adulthood (Hartley et al. 2011; Smith et al. 2012). Thorough assessment of the care needs driven by patient information will assist in the development of care guidelines for adults, including transitional care, and in providing optimal and holistic care for this vulnerable patient group. Hence, we performed a qualitative study to assess the full spectrum of the worries and the perceived healthcare needs in young adults with FXS, also including parents as representatives. Physical, psychological and socio-economical domains were discussed, and worries and needs were categorized according to the International Classification of Functioning and Disability (ICF) (WHO 2001) to provide a comprehensive interpretation. These patient-driven data were used to formulate recommendations for transitional and adult care, with the aim to improve care for adult patients with FXS.

## Methods

A qualitative study was designed using semi-structured focus groups and individual interviews to collect data on the worries and healthcare needs of young adult patients with FXS and parents of patients. Various domains were assessed, including medical, psychological and socio-economical domains (see Table 1).

**Table 1:** Abbreviated interview guide

Key questions: 1. What are your concerns about… 2. What are your care needs for…	Probes
Transition from pediatric to adult care?	Transition to adult healthcare, change in physician, transition to adult life, transition to work, independent living
Medical issues?	Symptoms, FXS-related care, medication, care consultations
Psychological and behavioral issues?	Sleeping problems, symptoms of ADHD, symptoms of ASD, depression, anxiety, aggression, self-injurious behavior
Social life?	Friendships, romantic relationships, family, loneliness, family planning, sexuality
Work and daily activities?	Work, school/daytime activities, independence, finances, daily living, planning
Paramedical issues?	Communication, nutrition, motor skills

### Patient Inclusion

For recruitment purposes, the study was advertised in the monthly newsletter of the Dutch FXS patient organization (Fragiele X Vereniging Nederland, http://www.fragielex.nl). Additionally, purposive sampling was performed, inviting three female patients. Recruited patients and parents were invited for focus groups or personal interviews which took place between June 2016 and April 2017. To be eligible for participation, patients had to be between 18 and 30 years of age and have a genetically confirmed diagnosis of FXS. The inclusion criterion for the parents was having a child with FXS between 18 and 30 years of age. Results of the genetic mutation analysis were not retrieved.

### Data Collection

The focus groups were held in person, each group with a duration of around 90 minutes. In addition to these focus groups, individual interviews were held in person or by phone. The focus groups and the individual interviews were performed by a representative of the patient foundation, a medical student (L.H.), an intellectual disability physician in training (M.R.), and an intellectual disability physician (A.E.). The semi-structured interviews contained questions about the worries and care needs on multiple domains during the transition phase (see Table 1). The focus groups and individual interviews were documented on video and/or audio recordings.

Both focus groups and individual interviews were held. Focus groups do not discriminate against people who cannot read or write and they can encourage participation from people unwilling to be interviewed on their own or who feel they have nothing to say. Individual interviews on the other hand represent the voices of people who possibly would be silenced in a focus group by group norms.

### Data Analysis

The recordings of the focus groups and individual interviews were transcribed verbatim using ATLAS.ti 8.0 qualitative software package (Atlas.ti 2017). The transcripts were reviewed and coded separately by two members of the research team (L.H. and M.R.). The individual codes were compared and harmonized, to reduce the influence of each individual researcher. Subsequently, the codes were organized into themes. The codes and the arising themes were continually discussed with a co-investigator (A.E.) to increase validity. In an additional meeting, these themes were discussed with experts on FXS (M.C. and B.D.) until consensus was reached.

The ICF was used to organize the emerging themes. The ICF is a classification of health and health-related domains and the World Health Organisation framework for measuring health and disability at both individual and population levels (WHO 2001). The ICF defines the following domains: impairment of body function and structures, activity limitations and participation restriction, and environmental factors. Results for males and females were analyzed separately because of the phenotypic differences of these subgroups (Hartley et al. 2011).

### Ethical Approval

A formal review and waiver was provided by the medical and ethical review committee (MEC) of the Erasmus University Medical Centre (ref. MEC-2016-532), in accordance with the 1964 Helsinki declaration and its later amendments or comparable ethical standards. Informed consent was obtained from all individual participants for cooperation and for video and/or audio recordings. For the individuals with FXS who took part in the study, consideration of mental competence was performed by a trained interviewer.

## Results

### Study Population

Thirty-eight participants were enrolled in the study, including thirty-three parents and 5 patients (see Tables 2, 3 for participant characteristics). One of the parents was a foster mother. Four focus groups were organized. Two focus groups consisted of parents of male patients aged 18 to 30 years, one focus group consisted of parents of female patients aged 18 to 30 years, and one focus group consisted of female patients aged 18 to 30 years. In the focus group of female patients, no parents were present. Eleven individual interviews were held, including seven by phone and four in person. One male and one female patient were individually interviewed. Five interviews were performed with parents of male patients and four interviews were performed with parents of female patients. All of the focus groups and interviews were recorded and transcribed anonymously. As no new codes and themes arose during analysis of the last 3 interviews, data saturation was considered to be achieved. Fifteen themes were identified after transcription and coding, and were classified in the domains of the ICF framework (Table 4). There were no themes that were discussed by parents or patients only. There were some themes that were more extensively discussed by parents, as stated in Table 4. There were no contradictory views or reports. Overlapping themes for males and females were anxiety, limited social skills, somatization, physical problems (e.g. fatigue, ear infections), difficulties with physical exercise, maintaining romantic and social relationships, lack of independence, parental stress, financial problems, and limited knowledge on FXS in healthcare providers. The results per ICF domain are more specifically discussed below.

**Table 2:** Male patient and parent characteristics

Characteristics	Focus group patients	Focus group parents	Interview patients	Interview parents	Total
Participants	n/a	15	1	5	21
Age of patients in years, mean (SD)	n/a	23.3 (4.9)	23	23.3 (3.8)	23.3 (4.7)
Cognitive level	All patients IQ < 70				

**Table 3:** Female patient and parent characteristics

Characteristics	Focus group patients	Focus group parents	Interview patients	Interview parents	Total
Participants	3	9	1	4	17
Age of patients in years, mean (SD)	22 (5.3)	25.6 (5.3)	19	24.3 (5.9)	24.2 (5.3)
Cognitive level	All patients IQ > 70				

**Table 4:** ICF categories, themes, and corresponding codes

ICF classification	Themes	Codes for males	Codes for females
Impairment of body functions and structures	Mental health problems	Anxiety, symptoms of autism, sleeping problems^b^, behavioral problems (aggression, self-injurious behavior, pica)^b^	Anxiety, symptoms of autism^b^, fatigue
	Visibility of disability	Not discussed	Disorder not obvious, causing misunderstanding and overestimation by environment
	Cognitive deficits	Problems with language and speech^b^	Learning disabilities^b^, dyscalculia^a^, need for extra support^b^, slower learning^b^, difficulties planning and organizing
	Family planning difficulties	Parental anxiety about reproduction^b^	Doubts about parenting capabilities, not wanting a child with FXS^a^
	Physical abnormalities	Abnormal pain perception, not capable to communicate complaints^b^, ear infections, clumsiness, somatizing^b^	Ear infections, clumsiness, somatizing
	Use of medication	Side effects of medication, wish to reduce medication, lack of experience in prescribing physicians^b^	Not discussed
Activity limitations and participation restriction	Limitation of independence	Complete dependency on others	Difficulties reaching independence, need of help from parents^a^, not having a driving license, vulnerability^b^
	Activity	Difficulty with physical exercise^b^, lack of exercise	Difficulty with physical exercise
	Difficulties in relationships	Trouble with initiating and maintaining social and romantic relationships, no need for friends, friendship initiated by parents^a^	Trouble with initiating and maintaining social and romantic relationships, loneliness, friendships initiated by parents^b^, vulnerable in relationships^b^
	Sexuality	Masturbation in public, self-stimulatory behavior, atypical sexual interests, inappropriate sexual behavior^b^	Not discussed
	Limitations with work and school	Limited availability of appropriate daytime occupation	Unable to work full time^b^, overestimation^b^, difficulties with finding an appropriate job, difficulties in relationships with co-workers^a^, apprehension about high workload^a^
Environmental factors	Parental stress	Much time spent on assistance and administration, worries about independency and vulnerability of children, need of more attention to parental worries and needs, worries about the future: care after parents decease^b^	Much time spent on assistance and administration, worries about independency and vulnerability of children, need of more attention to parental worries and needs,^,^ worries about the future: care after parents decease^b^
	Problems with the financial system	Worries about healthcare cuts, much time spend on financial administration^b^	Worries about healthcare cuts, eligibility for social security benefits, much time spent on financial administration^b^
	Unavailability of adequate care and support	Knowledge FXS absent in healthcare providers, need for help with administration and applications for financial support, need for adequate housing, problems with finding appropriate care^b^	Knowledge FXS absent in healthcare providers^b^, need for help with administration and applications for financial support, need for adequate housing, problems with finding appropriate care^b^
	Problems during transition process	Unsuccessful transition, not ready for transition, missing support of caregivers^b^	Unsuccessful transition, not ready for transition^b^

^a^Mainly discussed by patients

^b^Mainly discussed by parents

### Impairment of Body and Functions and Structures

#### Mental Health Problems

Concerns about behavioral problems such as aggression, self-injurious behavior and pica were extensively discussed by parents of male patients. Aggression was thought to have multiple causes, such as being overestimated, anxiety, frustration, sensory overload, and as an expression of underlying physical problems. Self-injurious behavior occurred frequently and worsened in moments with high anxiety and/or arousal. Hand and arm biting, tearing off toenails, beating and scratching themselves to the point of bleeding were mentioned. “The behavioral problems have existed since his childhood, he was always very aggressive towards his little brother, […] he can’t stand a lot of noise, he becomes frustrated and starts ripping his clothes and peeing himself” (Parent 1). Most parents felt helpless in coping with these behavioral problems. In general, behavioral problems were perceived to decrease after adolescence.

High levels of stress and anxiety were mentioned in almost every focus group and interview of male as well as female patients. “He is ruled by his anxiety and phobias, […] our biggest fear is that he is not capable of overcoming his anxiety disorder and that it becomes his downfall” (Parent 2). Pain, unknown surroundings, doctors, dentists and medical treatment were mentioned multiple times as potential triggers. Problems with sleeping and symptoms of autism, such as social deficits and rigidity, were also discussed in all focus groups and interviews.

In addition to symptoms of anxiety and ASD, the female patients themselves encountered problems with concentration, attention, and fatigue. In these females, chronic worries were present on many domains, leading to sleeping problems. “I sometimes have trouble with sleeping, then I mull about work and go over the whole day in my head” (Female patient 1).

#### Visibility of Disability

Female patients experienced that their normal appearance lead to overestimation of their abilities and intelligence. They continuously needed to explain their disabilities and were afraid of disappointing others. “Because people can’t see that I have FXS, I constantly need to explain it. It’s important that people understand, so that they take it into account” (Female patient 2).

#### Cognitive Deficits

As reported by parents, all the male patients of this study had an ID and required daily support and care. Problems with language and speech were mentioned. Some used simple sign language to express themselves, and visual tools were used to help with the communication and planning.

The female patients had no or only a mild ID as reported by parents, and needed help in specific areas, such as school, work and planning. All females reported problems with math and problems with planning and organization.

#### Physical Problems

Not many medical problems were mentioned for the male patients. Abnormal pain perception was reported often, which was described by parents as a decreased sensitivity to pain. Pain or discomfort was often not directly indicated by patients, but manifested itself by behavioral changes. Almost all parents were afraid that because of this abnormal pain perception and expression, injuries or diseases would go unnoticed, also in adulthood. “My son can talk well and report what he wants. Yet, he never points out having pain, it’s puzzling…” (Parent 3). Parents expressed the need of regular medical checkups and were afraid that physicians would not perform sufficiently thorough checkups because of the minimal complaints.

Problems that were mentioned in both male and female groups were recurrent ear infections, epilepsy, and problems with motor skills, frequently described as clumsiness. Parents in both groups also discussed the presence of somatization, the presentation of physical complaints as an expression of stress or anxiety. “When tension runs high she always gets abdominal pains” (Parent 4).

#### Family Planning Difficulties

Female patients were concerned about reproduction due to the heritability of the disorder. “I would like to have children. But having children is difficult; they could also have FXS, and then you will get in all sorts of procedures, which is emotionally difficult. I would think twice” (Female patient 1). Parents were also concerned about reproduction due to the heritability of the disorder, and also expressed doubts on parenting skills. “When our son turned 18 we spoke with him about children and we agreed that sterilization was for the best. Having grandchildren is very nice but it would be a disaster if he would be responsible for a child” (Parent 3).

#### Use of Medication

Many of the male patients were using medication for behavioral problems and anxiety. Parents worried about the lack of expertise in physicians. They expressed the need for more knowledge on medication for anxiety, behavioral problems and depression. “There is too little expertise when it comes to medication, we searched the last 23–25 year for the right guidance (…) It’s kind of a trial and error system” (Parent 5). Problems with adverse effects like weight gain and deterioration of the gross motor skills were also mentioned. Parents wanted to discontinue or reduce psychotropic medication but found this to be difficult and not always feasible. In some cases parents missed support of their physician in the attempt to reduce the medication.

### Activity Limitations and Participation Restriction

#### Limitation of Independence

Most of the female patients had trouble with reaching independence. They needed help from their parents with finances and administration and reported limitations in planning and organizational skills. Some of the female patients were capable of living independently but required assistance for housekeeping, finances, planning and organization. “My daughter lives on her own but she is not independent. My husband and I do everything for her: the finances, cooking and cleaning. Sometimes I feel like I let a 14 year old move out of the house” (Parent 6).

#### Activity

Patients and parents in both groups mentioned concerns about sports and the level of physical exercise. Some of the patients had trouble with physical exercise due to the problems with motor skills. Parents stressed the importance of physical exercise and worried that their children were not getting enough exercise in the occupational and living facilities. “When my son lived nearby, we went swimming once every 2 weeks and we would go to the gym. Since he moved to another facility this is no longer an option, now he has no physical activity at all” (Parent 7).

#### Difficulties in Relationships

Social deficits were extensively discussed both in the female focus groups and interviews as well as in the male focus groups and interviews. The impact of the social problems was large and caused patients and parents a great deal of worries. The largest problem was starting and maintaining relationships, both socially and romantically. “Our son doesn’t initiate any kind of relationship, he is always alone and has no need for friends” (Parent 7).

Female patients mentioned that they lacked social skills and had problems initiating and maintaining friendships. Also, they found it difficult to determine the appropriate time and approach to inform people about FXS. “I dated some guys, just to get to know each other, but then… I have never told a boy about FXS, it doesn’t reach that point, we are never on the same page” (Female patient 1). All of the female patients mentioned that they were not ready for a romantic or sexual relationship.

Parents of both groups were concerned about the loneliness and limited social life of their child. Parents helped their child by facilitating and maintaining friendships for them. They worried about the lack of need for friendships in their child. “I always need to remind her to contact her friend, suggest that they meet and go do something fun together. If I don’t do this, it doesn’t happen at all” (Parent 8).

#### Sexuality

Regarding sexuality there were many problems mentioned in the male patient focus groups and interviews. Parents worried about excessive self-stimulatory behavior, masturbation in public and the misunderstanding of others of this subject. Problems around sexualization of feces and other atypical sexual interests were also discussed. Some of the male patients did not show any interest in sexuality. In case of no sexual interest at all, parents of male patients were wondering if they should familiarize their children with masturbation. Some parents reported excessive masturbation in male patients, also as a means to relieve tension. All parents worried about the sexual vulnerability of their child. “I am afraid that someday he will run in to the wrong person and that they take advantage of him” (Parent 9).

#### Limitations with Work and School

All parents expressed the need for a sheltered workplace with appropriate support, content and guidance, and reported difficulties in finding this. Parents of female patients worried about finding an appropriate job for their daughter.

Female patients worried about finding a workplace with an appropriate workload, as working full time was generally perceived as challenging and overwhelming. Also, the overestimation of their abilities by others, as well as by themselves, created stress. Social interactions with co-workers and team work was also reported to be stressful. “Work can be difficult for my daughter, it depends on which boss is working. She really is in need of a good job coach, someone who knows her strengths and weaknesses” (Parent 6).

### Environmental Factors

#### Problems with the Financial System

Parents were worried about recent financial cuts in the Dutch healthcare system. The administration necessary for applying and re-applying for a personal budget was discussed to be very complex and frustrating, and parents felt distrusted by government employees.

Parents of the female patients also expressed concerns that their daughter did not qualify for a personal budget, social security benefits, or reimbursement for the needed care, as females were often deemed ineligible due to normal intelligence levels. To qualify for social security benefits or personal budget the female patients often needed an additional diagnosis from a psychiatrist. “There are no social benefits available for my daughter. She is too smart but on the other hand she is not able to be on her own. We hope to get a personal budget. For now she slips through the cracks” (Parent 8).

#### Unavailability of Adequate Care and Support

Parents encountered a lack of knowledge of FXS in physicians and other healthcare providers. “As a parent, you always need to explain what FXS is, even to some physicians. It’s so typical for FXS. When you say my daughter has Down syndrome, everybody knows what you’re talking about” (Parent 9). Some parents carried along information leaflets for the physician to read. Parents also had the feeling that their child had received inadequate care when parents were not involved. Parents expressed the need of a multidisciplinary treatment center for adult patients with FXS, that could provide information for local healthcare providers and living facilities. Parents also indicated a lack of appropriate living facilities and healthcare for females.

#### Parental Stress

Parents expressed that they experienced a great amount of pressure in being solely responsible for the organization and administration of their child’s life. They also felt alone in the coordination of care, and missed support in the care system as well as in their direct environment.

Parents of both groups worried about the future, especially about the moment when they would be too old or deceased. “I worry about the future, what will happen when I can’t coordinate every aspect of the care for my son. I am afraid that the helpers in the living facility will become neglectful” (Parent 1).

#### Problems During Transition Process

Not many patients were under treatment by a pediatrician during the transitional age, and around the age of 18 most patient were in good physical health and under supervision of a general practitioner. Parents found it difficult that medical specialists treated their child as a normal functioning adult and were not aware of the FXS. “My child is 21 and has a mental age of 13–14 years […] So why does she have to transition into adult care, when she has the cognitive level of a child?” (Parent 4). Some parents experienced the transition to adult care as insufficient, because no referral was performed and no communication between pediatric and adult physicians took place. Parents also remarked that they had to figure everything out by themselves and missed support. Parents of patients who were under treatment by an ID physician [a relatively new medical specialty in the Netherlands (Ewals 2014)] were generally satisfied with this care and the FXS-specific expertise.

## Discussion

The patient-driven, qualitative data yielded in the current study revealed a myriad of needs on physical and mental health domains, activities and participation, and environmental domains for young adult males and females with FXS. Our study shows that the burden of FXS is not limited to physical and mental health issues and differs for men and women, enabling us to formulate recommendations regarding the organization and the content of care.

### Mental Health

For males with FXS, the prevailing theme of anxiety concurs with reports in the literature: 70–86.2% of males with FXS meet criteria for an anxiety disorder (Bailey et al. 2008; Cordeiro et al. 2011; Wheeler et al. 2014). Parents reported worsening of aggression and self-injurious behavior during episodes of high anxiety and/or arousal. This confirms observations that sensory issues and hyperactivity were significant predictors of the frequency of aggression, while sensory issues and anxiety were predictive of the severity of aggression (Wheeler et al. 2016). The symptoms of autism that remained a concern of parents of male patients, confirms that autistic features persist over time in FXS (Smith et al. 2012) and necessitates appropriate care also in adulthood. Males with FXS and ASD tend to have lower IQ scores, poorer adaptive skills, and less advanced language skills than individuals with FXS without ASD (Bailey et al. 1998; Philofsky et al. 2004), perhaps also contributing to the concerns of parents.

In females with FXS, as reported by patients as well as parents, anxiety disorders were also a common concern, confirming observations that 76.9% of females (ages 5.0–33.3 years) met criteria for an anxiety disorder, with many more showing subclinical symptoms of anxiety (Cordeiro et al. 2011). The discussed limitations in attention, planning and organizing confirm the reported impairments in executive functioning in this group (Bennetto et al. 2001). ASD and limited social skills were also mentioned. Although 16–20% of females with FXS meet diagnostic criteria for autism (Hall et al. 2008; Kaufmann et al. 2017), social skill impairments in females with FXS could also be related to (a combination of) underlying (social) anxiety, attentional deficits, low self-esteem, and/or executive dysfunction, and should be further investigated.

The tendency to experience and communicate psychological distress in the form of physical symptoms, henceforth called somatization, was discussed as a concern by parents for males as well as females with FXS, and has not been previously described in FXS. Although there is very limited evidence on psychological defense mechanisms in general, or on somatization in intellectual disability, it seems reasonable to assume that cognitive impairments, anxiety, and limited coping and defense mechanisms may account for the seemingly frequent occurrence of somatoform complaints in these patients. Diagnosing somatization must be performed prudently after thorough physical evaluation and professionals must remain aware of abnormal pain presentation and atypical presentation of physical complaints in patients with FXS and ID in general.

### Sexuality, Romantic Relationships, Family Planning

Parents of male patients discussed public masturbation, other inappropriate self-stimulatory behavior, and atypical sexual interests. Parents had many questions on sexual development but could not find expertise on this domain. Although the American National Fragile X Foundation has published an overview for parents on how to approach sexuality issues in FXS (Bergner et al. 2012), such an overview is not yet available in the Netherlands.

Initiating and maintaining social relationships was difficult for female patients as well as for the males. In general, parents of male patients agreed this was not experienced as a problem by their sons. Female patients, however, suffered of their limitations in social and romantic relationships, causing social anxiety and loneliness. Hartley et al. (2011) found that only 60% of adult females had considerable friendships and only one-third of the women with FXS lived independently, often with a spouse or romantic partner (Hartley et al. 2011), suggesting that support in initiating and maintaining social and romantic relationships is needed for these vulnerable women.

### Physical Health

Although literature and guidelines focus mostly on somatic issues in children (Hersh et al. 2011), these issues persist and may worsen in adulthood.

Abnormal pain presentation was a big concern for parents of males. This abnormal pain perception however, could be better perceived as an abnormal pain presentation response (Foley and McCutcheon 2004), and De Knegt et al. even hypothesize that patients with FXS may suffer from an increase in pain perception (de Knegt and Scherder 2011). Noticing behavioral changes consistent with having pain is often difficult, as these changes may be subtle or different from an expected response (Foley and McCutcheon 2004), and the international guideline of the National Institute for Health and Care Excellence (NICE) (NICE 2015) emphasizes the need to rule out health problems as a cause of challenging behavior.

Physical health problems such as ear infections, epilepsy, and motor problems were noted as concerns in both patient groups. Other known physical health features of male patients with FXS, such as mitral valve anomalies, joint hypermobility or refractive eye problems were not mentioned by parents, which can be explained by the low frequency and disease burden of these features in adults (Ciaccio et al. 2017). Lack of awareness might also play a role. Features such as neurological symptoms (seizures and movement disorders), gastrointestinal symptoms, hypertension and obesity are frequent in adults with FXS (Utari et al. 2010) but were not mentioned as a concern. Recommendations for monitoring physical health, including motivational support for lifestyle-related issues such as achieving and maintaining a healthy body weight, refraining from excessive alcohol consumption, smoking cessation, searching opportunities for physical activities are summarized in Table 5.

**Table 5:** Clinical recommendations for transitional and adult care, based on literature and findings of our study

ICF domain	Screen males for:	Screen females for:	Provide
Impairment of body functions and structures	*Physical health* • Neurological problems • Gastrointestinal problems • Obesity • Hypertension • Heart-problems • Use of medication and side effects • Abnormal pain perception and presentation • Fatigue • Contraception	*Physical health* • Gastrointestinal problems • Obesity • Hypertension • Use of medication and side effects • Fatigue • Questions on menstruation regulation, contraception, family planning	Yearly screening by easily accessible FXS-expert, screening on all domains, with organ-specific care when indicated Motivational support e.g. for life-style related issues Close communication between GP, care professionals and FXS-experts
	*Mental health* • Anxiety • Symptoms of ASD • Symptoms of ADHD • Depressive symptoms • Cognitive and/or adaptive functioning • Somatization	*Mental health* • Anxiety • Symptoms of ASD • Symptoms of ADHD • Depressive symptoms • Somatization • Cognitive functioning • Executive functioning impairments	Yearly screening for mental health issues Episodic (neuro)psychological evaluation and psycho-education, e.g. at least once per 5 years When indicated: interventions with (non-verbal) behavioral therapies and psychotropic medication Peer groups
Activity limitations and participation restriction	*Participation and work* • Self-management • Participation • Adequate work or daytime occupation • Friendships • Recreation, exercise *Relationships and sexuality* • (Romantic) relationships • Sexual development and education • Inappropriate sexual behavior	*Participation and work* • Self-management • Participation • Adequate work or daytime occupation • Friendships • Recreation, exercise • Social vulnerability *Relationships and sexuality* • (Romantic) relationships • Sexual development and education • Family planning	Involvement of a social worker to optimize socialization and independence Information and advice for employers and social services Involvement of a job coach for appropriate daytime occupation Discuss issues separately with patients as well as parents/caregivers
Environmental factors	Administrative burden Financial problems Living circumstances Information and knowledge on FXS, also for environment Parental stress Transitional care	Administrative burden Financial problems Living circumstances Information and knowledge on FXS, also in environment Parental stress Transitional care	Involvement of a social worker and/or coach Information on patient organizations Information material Address parental concerns and increase support in daily life through GP, parent support groups, social services or psychologist Involve both parents and young adults in transition process Designated coordinator of the transition process Plan the transitional process timely, preferably at the beginning of adolescence, in collaboration with a FXS expertise center A FXS expertise center should be easily accessible for the patients, as well as for local healthcare providers Patient organizations could facilitate knowledge on care infrastructure, and help provide information to patients, caregivers, employers, and others

### Use of Medication

For males, many concerns arose about the use of medication. Parents discussed that psychopharmacological drug treatments were not evidence-based, and were generally based on a trial and error system. Indeed, data from Bailey et al. showed that approximately 10%–20% of parents thought that use of psychopharmacological treatment was not helpful for the behavioral problems of their son or daughter with FXS, whereas approximately less than one-third felt the medication was helping significantly (Bailey et al. 2012). Although in recent years progress has been made towards finding more targeted treatment options (Berry-Kravis et al. 2017; Zeidler et al. 2017), there is insufficient evidence-based medicine for FXS. In addition to identifying disease-modifying drugs, randomized controlled trials should be performed for generally prescribed psychotropics such as antipsychotics, anti-depressants, and stimulants to improve management and guidelines for FXS. In the meantime, treatments should be properly indicated, explained, and documented, applying thorough evaluation methods.

### Activities and Participation

During adolescence, individuals try to achieve emotional, personal, and financial independence from their parents. However, male patients and most female patients still relied on help from their parents for all of these domains during this period. For women with FXS, Hartley et al. showed that higher age and the ability to interact appropriately were predictive of a higher level of independence in several dimensions of adult life (Hartley et al. 2011), implicating that the transitional period can be protracted, and that support to achieve independency should be maintained in adulthood. Information and appropriate support in the work and living environment could alleviate the stress caused by limited independency. Additionally, peer groups can have a significant positive impact, as has been mentioned by youngsters with Neurofibromatosis Type 1 (Rietman et al. 2018). Lastly, inclusion in appropriate physical activities will improve participation as well as physical and mental health.

### Environmental Factors

Parents expressed a lack of knowledge about FXS in most physicians and other healthcare providers, and lack of health evaluations. This is confirmed by a recent study by Visootsak et al., which showed that their patients had not received appropriate FXS-specific care prior to visiting their Fragile X clinic, leading to underdiagnosis and undertreatment (Visootsak et al. 2016). Our study confirms the need for a multidisciplinary team and approach, also for adults with FXS. In the Netherlands, an ID physician would be a key figure in such a team, because of their expertise on ID, genetic neurocognitive disorders and their holistic approach on all ICF domains. Unfortunately, this medical specialty is unavailable elsewhere, and a multidisciplinary team should consist of FXS-specific expertise in internal medicine, psychiatry, psychology, and social work (see Table 5). Additionally, patient organisations are a very important source of information, and such organisations should empower patients to find appropriate information, care and support, including parent and peer support groups.

### Parental Stress

Parents of both males and females experienced many concerns and high stress levels, as has also been described in other studies (Hartley et al. 2012; Lewis et al. 2006; McCarthy et al. 2006). Parents worried about the future, about the lack of independence and need for constant guidance of their child. Concerns about their naivety and vulnerability were also frequently reported. Parents also experienced inadequate support in their environment, for instance of family, social networks and employers. Parents of both groups expressed the need for professional support for themselves to cope with their concerns. Care providers as well as patient organizations should provide information on, and support for, parental stress.

### Transitional Care

Our study shows different themes for parent- and patient-reported worries, which underline that both parents and young adults should be involved in the transitional process (Lotstein et al. 2009), although care should be taken to optimize autonomy for female patients and mildly affected male patients in particular. In addition, parents are often concerned about the lack of information concerning transition of care, and they expressed the wish for earlier and better coordinated transition planning (Reiss et al. 2005).

Only a few patients in this study experienced a formal transition from pediatric to adult care. Patients with FXS, similar to other neurodevelopmental disorders, are likely to experience problems during transition to adult health care. Reported risk factors may also apply to many patients with FXS, such as living independently from parents, male gender, lower family income, greater travel distance to adult specialized clinic (Goossens et al. 2016), epilepsy (Geerlings et al. 2015), ASD (Friedman et al. 2013), and a lack of knowledge and expertise of healthcare providers on pediatric neurological and neuropsychiatric diseases (Camfield and Camfield 2011).

Although attention for the transition of care from pediatric to adult health services has increased, a recent review of the Cochrane Library showed limited evidence on the efficacy of interventions to improve the transition from pediatric to adult health services (Campbell et al. 2016). However, the need for evidence-based transitional care and interventions is urgent and is emphasized in our study. Also, more attention to the holistic process of moving to adulthood and independence, instead of solely focusing on transfer of medical care, is indispensable (Kirk 2008).

### Implications and Recommendations

Our results underline the burden of FXS for both male and female patients, and the need for periodic multidisciplinary care, screening for problems on all ICF domains. As the transitional age and young adulthood is a complex phase in life, we advise yearly screening. As the physical and neuropsychiatric phenotype persists, and cognitive ageing in FXS has been reported at a relatively early age (Schneider et al. 2013), this yearly screening should be continued throughout the lifetime of adults with FXS, including neuropsychological evaluation at least once every 5 years. The need for gender specific care is emphasized by this study, as male and female patients address different worries and needs, although on similar domains. Suggestions for clinical practice in both patient groups are summarized in Table 5.

### Strengths and Limitations

An important strength of the present study is the use of both patient- and parent-driven data, in focus groups and individual, semi-structured interviews. The use of focus groups as a method to discuss the worries and needs of the parents and patients has some limitations, such as possible limitations in sharing sensitive issues. However, because we also used individual interviews and reached data saturation, most issues appeared to be addressed.

Most patients and parents were members of the Dutch Foundation for FXS patients, which may have resulted into a biased sample where parents were relatively well-informed and assertive. Purposive sampling of female patients may have resulted in a relatively severe phenotype of these participants. Premutation carrier status of parents was not known; as FXS premutations are associated with anxiety (Bourgeois et al. 2011; Roberts et al. 2009), this may have influenced the data.

Because of the low mean IQ and comorbid ASD and/or anxiety in men with FXS, it was difficult to include male patients who could communicate their concerns and care needs, and more patient-driven information using novel interview methods is needed to improve our understanding of concerns and needs. Recruitment of female patients for this study was difficult, as not many are involved with the Dutch Foundation for FXS patients, and perhaps also due to personality traits such as shyness and anxiety. This recruitment problem is exemplary for the care gap in the female patient population, and our findings underscore the burden of FXS in women and the importance of diagnosis and appropriate care. For both male and female patients, recruitment for interviews was limited. For this reason, caregivers were included as representatives of these patients. The difficulties in recruitment underline the need for national and international collaboration for shared data collection and research activities.

AD(H)D is a common diagnosis in male and female patients with FXS (Sullivan et al. 2006). In our study, this subject was not mentioned by patients or parents. This could be due to decreasing ADHD-symptoms in adults or due to underdiagnosis, as limitations in executive functions were frequently mentioned which often co-occur with ADHD. Another explanation is that in patients with ASD, an additional diagnosis of ADHD was not allowed by the previous Diagnostic and Statistical Manual of Mental Disorders, fourth edition (DSM-IV). In the DSM, fifth edition (DSM-V) this is no longer an exclusion criterion. This emphasizes the need for further research into the adult neuropsychological phenotype, as well as the developmental trajectories and aging process for adolescents and young adults with FXS.

Finally, as the Netherlands is a relatively small and a high-income country in Western Europe, generalization of current findings is limited to similar countries with respect to care infrastructure, expertise and finances. We expect an even larger care gap in lower income countries. However, the U.S. and Canada already have some well-functioning multidisciplinary centers for adults with FXS, which could function as a model for the Netherlands and others.

## Conclusion

This qualitative study yielded a broad and unique insight in the worries and needs of young adult patients with FXS and their parents, on physical and mental health, activities and participation and environmental domains. Limitations in transitional and adult care and expertise became apparent, underlining the need for syndrome-specific care in FXS, including a multidisciplinary and family-centered approach, with attention for variability and differences in needs in patients due to the level of cognitive functioning, psychiatric comorbidity and gender differences. These patient- and parent-driven data support the impact of FXS on families as described in 1986 by John M. Opitz:

And then as always, one stops to recollect with total astonishment and great reverence the massive burden of pain carried so patiently by the mothers, fathers, sibs, grandparents and the many others involved so closely on a daily basis with the apparent failure, defect, handicap, disability, and disappointment in the many thousands of Martin-Bell syndrome families throughout the world (Opitz 1986).
